# Development of a questionnaire and screening model for lipedema

**DOI:** 10.1590/1677-5449.200114

**Published:** 2020-12-11

**Authors:** Alexandre Campos Moraes Amato, Fernando Campos Moraes Amato, Daniel Augusto Benitti, Lorena Guimarães Lima Amato

**Affiliations:** 1 Universidade Santo Amaro – UNISA, São Paulo, SP, Brasil.; 2 Amato - Instituto de Medicina Avançada, São Paulo, SP, Brasil.; 3 Medical Valens Center, Campinas, SP, Brasil.; 4 Universidade Nove de Julho – UNINOVE, São Paulo, SP, Brasil.

**Keywords:** questionnaires, lipedema, obesity, lymphedema

## Abstract

**Background:**

Lipedema is greatly underdiagnosed and there is a lack of low-cost tools to facilitate diagnostic. We created a lipedema screening questionnaire based on a questionnaire for assessing symptoms.

**Objectives:**

The study objectives were to identify relevant clinical questions, develop a screening questionnaire, and construct a model for predicting lipedema.

**Method:**

A simplified questionnaire was constructed and administered to a sample of patients with and without lipedema and then the probability of correct diagnosis was analyzed.

**Results:**

All 109 patients who answered the questionnaire were female and all of them understood the questions. A predictive model using individual question scores achieved an excellent probability of correct diagnosis, at 91.2%, and a predictive model based on total score also achieved a good probability of correct diagnosis, at 86.15%.

**Conclusions:**

The lipedema screening questionnaire is a practical instrument that is quick and easy to administer and can be used with our population for identification of possible lipedema patients, raising the level of suspicion when taking a patient’s history and conducting a physical examination.

## INTRODUCTION

Lipedema has been described in Brazil by Moraes, who refers to it as *lipofilia membralis*.^1^ It is characterized by abnormal bilateral build-up of fat in the gluteus, legs, and upper limbs that may be accompanied by orthostatic edema in postpubertal women.^2^ Anecdotal cases in men have also been described.^3^ While the pathophysiology of lipedema is still little understood, it has a high prevalence in the population, explained by its genetic aspect and its hormonal influence on cyclical inflammatory symptoms.^2^ It has been described as a low-level inflammatory process involving the lymphatic and adipose system,^2^ which is supported by a recent study by Wanshu Ma et al.^4^ These characteristics provoke typical symptoms, which are very often interpreted incorrectly, in addition to the build-up of fat. As a result, although lipedema is actually a distinct condition, it is often confused with more frequently diagnosed diseases, such as obesity and lymphedema.^5^^,^^6^ Lipedema is a disease with high prevalence, but low recognition and diagnosis, which is frequently underdiagnosed or confused with gynecoid obesity, lymphedema, or even venous insufficiency.^2^ Conservative estimates of the prevalence of lipedema in the general population range from 0.06 to 11%.^5^^,^^7^

Diagnosis of lipedema is essentially clinical, defined by symmetrically disproportionate build-up of fat in the lower limbs combined with complaints of orthostatic edema,^5^ with pain often present. The feet are not affected by the increase in size, except in advanced stages of lipolymphedema, in which edema of the feet is secondary to lymphatic insufficiency that is not present in earlier stages.^8^^,^^9^ Edema that does not involve the feet is an important sign for distinguishing lipedema from common obesity and lymphedema. Since there are no specific laboratory test markers of lipedema, with the exception of the promise shown recently by platelet factor 4,^4^ clinical diagnosis requires a high level of suspicion on the part of the treating physician. A questionnaire specifically for lipedema was developed in Germany^10^ and translated for Brazil by Amato et al.^11^ It is based on a quality of life questionnaire for patients with lymphatic diseases^11^^,^^12^ and was adapted to include 15 self-report criteria rated on an analog scale from 0 to 10. It is considered a lipedema symptoms scale and has not been validated as diagnostic or screening tool.

In view of the poor availability of objective instruments for screening for lipedema in Brazil and worldwide and considering that data on quality of life are important for selection and for interpretation of clinical progress, this study was conducted with the objective of identifying relevant questions and creating a mathematical prediction model, with the intention of raising the pre-assessment level of suspicion.

## METHODS

The process of identification and simplification of relevant questions suitable for self-report administration began by assessing the Portuguese translation of the symptoms questionnaire in its adapted form (Table 1). This questionnaire was converted into an on-line digital version using secure software appropriate for development and analysis of questionnaires (SurveyMonkey, California, United States) and was administered to 109 volunteers, with or without a prior lipedema diagnosis made during a medical consultation, but self-reported on the questionnaire. The study was approved by Ethical Committee (number 3.268.401).

**Table 1:** Final version of the lipedema screening questionnaire (translated from the Portuguese original that was administered).

Lipedema screening questionnaire			
Question	Responses	Score	p
Do you feel that there is something wrong with your legs, but don’t know what it is? (p1)	Yes, my legs are big, they look like posts/tree trunks and I have fat ankles.	3	< 0.001
	Yes, my legs are bigger (comparatively) than the rest of my body.	2	
	Yes, my legs are big and proportional to the rest of my body.	1	
	No, my legs are fine.	0	
Is the lower part of your body larger and disproportional compared to your trunk/upper body? (p2)	Yes, the bottom part of my body is clearly disproportional to my upper body/trunk. My pants are at least 3 sizes larger than my blouse size.	2	< 0.001
	Yes, my lower body is discretely disproportional to my trunk/upper body. My pants are 1 to 2 sizes larger than my blouse size.	1	
	No, my upper body/trunk is larger than the bottom half of my body. My blouse size is larger than my pants size.	0	
	No, the upper and lower parts of my body are proportional. My blouse size and pants size are the same.	0	
Do you have problems losing weight, particularly from the lower part of your body? (p3)	Yes, I can’t lose weight whatever I do, especially from my thighs/legs, hips, and/or arms, which just seem to get bigger.	2	< 0.001
	Yes, I try a lot, but only seem to lose weight from my trunk/upper body, often except for my arms.	1	
	No, with dieting and exercise I can manage to lose weight. Weight/fat seems to come off proportionally from my whole body.	0	
	No, I don’t have a weight problem or find it difficult to lose weight. My weight is normally stable.	0	
During puberty, did you put on weight, primarily on the thighs/legs, hips, buttocks, or arms? (p4)	Yes, I put on lots of weight during puberty, especially on my thighs and legs, hips, buttocks, and/or arms.	2	< 0.001
	Yes, I put on some weight during puberty. My thighs/legs, hips, buttocks, and/or arms seemed to get bigger than the other parts of my body.	1	
	No, I put on some weight during puberty, which was distributed around my whole body and not just my thighs/legs, hips, buttocks, and arms.	0	
	No, I put on a little weight, but lost it easily with dieting and exercise.	0	
	No, I didn’t put on weight. My weight was very stable during puberty.	0	
During or soon after pregnancy/breastfeeding, did you put on weight or notice changes to your thighs/legs, hips, buttocks, and/or arms? (p5)	Yes, I put on a lot of weight (> 23kg), mostly on my thighs/legs, hips, buttocks, and/or arms during or soon after pregnancy and/or breastfeeding.	2	0.148
	Yes, I put on 16-23kg, some on the thighs/legs, hips, buttocks, and/or arms soon after pregnancy and/or breastfeeding.	1	
	No, I put on weight normally (11-15kg), I didn’t put on extra weight on my thighs/legs, hips, buttocks, and/or arms during or soon after pregnancy and/or breastfeeding.	0	
	No, I put on less weight than expected or lost weight during or soon after pregnancy and/or breastfeeding.	0	
	Not applicable, I have never been pregnant.	0	
During the menopause, did you put on weight or notice changes to your thighs/legs, hips, buttocks, and/or arms? (p6)	Yes, I put on a lot of weight, and my thighs/legs, hips, buttocks, and/or arms got bigger.	2	-
	Yes, I put on weight, and my thighs/legs, hips, buttocks, and/or arms got a bit bigger.	1	
	Not really, I put on some weight, but it was equally distributed around my whole body or on my belly.	0	
	No, I didn’t put on weight.	0	
	Not applicable, I haven’t gone through the menopause yet.	0	
Do your legs hurt? (p7)	Yes, my legs are very sensitive. They’re painful or I feel like they’re burning even when they aren’t touched.	3	< 0.001
	Yes, my legs are painful and uncomfortable with any kind of contact.	2	
	Sometimes, my legs hurt if pressed or if I spend a long time standing up.	1	
	No, my legs don’t hurt.	0	
Do you have swollen legs? (p8)	Yes, my legs seem to be swollen all the time. It gets worse in the heat and when its humid and raising them doesn’t help with the swelling. Both legs swell up equally.	2	0.001
	Yes, my legs often feel swollen, but raising them sometimes helps. Both legs gets swollen.	1	
	No, my legs practically don’t swell up, swell up a little when it’s hot or humid, or only after a plane journey or when I have my period, but they quickly go back to normal size, especially if I raise them. And/or only one of my legs swells up.	0	
	No, I rarely feel my legs are swollen.	0	
Do your legs or arms bruise easily? (p9)	Yes, my legs and arms bruise very easily, I don’t even know how I manage to bruise them.	2	0.002
	Yes, my legs and arms sometimes bruise, with minimal contact.	1	
	I don’t bruise easily.	0	

### Diagnosis of lipedema

Diagnosis of lipedema is still eminently clinical. An experienced assessor used the following criteria to classify members of the group with the disease: suggestive clinical history in postpubertal women; symmetrical bilateral deposits of fat below the hips, but not involving the feet (negative Stemmer sign); non-depressible edema (negative Godet sign), resistant to raising the limbs; areas affected painful and sensitive to palpation; and increased capillary fragility, with spontaneous ecchymosis.^2^^,^^3^^,^^5^^,^^6^^,^^8^^,^^9^

### Administration of questionnaires

During the on-line validation of the questionnaire, volunteers from a specific group of women with and without lipedema agreed to answer the questionnaire in electronic format, without external help, and free to fill in all data as they saw fit. The sampling technique was non-probabilistic by convenience, and subjects were invited to take part after assessment at a Lymphedema and Angiodysplasia Clinic. Women over the age of 18 years seen for any type of complaint were recruited. Men were excluded, as were women who did not sign the consent form, had serious arterial or venous conditions, or could not speak or understand Portuguese.

The primary objective of this study was to develop a simple questionnaire that would be quick to answer, for screening for lipedema. A secondary objective was to construct a mathematical model for predicting lipedema based on the questionnaire.

### Statistical analysis

Statistical analysis was conducted after the consistency of the data had been checked manually. Descriptive statistics and frequencies were calculated. Correlations between questionnaire variables were assessed using Spearman correlations and the Shapiro-Wilk test. Correlations with total scores were assessed using Pearson’s correlation coefficients. Statistical analysis was performed using Student’s *t* test. We adopted a p < 0.05 level of statistical significance for the correlations. The software used for data analysis was Excel (Microsoft, Novo Mexico, United States) and Wizard 1.9.40 (Evan Miller, Massachusetts, United States).

This study complies with the National Health Council (Conselho Nacional de Saúde) standards set out in resolution 196/96 regulating research involving human beings. It also adheres to the Helsinki Declaration and was approved by the Plataforma Brasil Research Ethics Committee, under protocol CAAE: 09590919.6.0000.0081.

## RESULTS

One hundred and nine women with a known prior diagnosis completed the questionnaire. All interviewees understood the questions. None of the questions were considered non-applicable. The proportion between patients with and without lipedema was uniform (z score, p = 0.389) (Table 2).

**Table 2:** Characteristics of the sample.

	**Lipedema**	**No lipedema**	**Chi-square**	**Result**
n	59	50	p = 0,389	Uniform
Age (years)	42 (19-77)	45.7 (24-79)	p = 0.165	Independent
BMI (kg/cm^2^)	29.68 (20.4-46.2)	29.1 (20.4-45.6)	p = 0.312	Independent
Height (cm)	163.7 (148-183)	165 (139-183)	p = 0.113	Independent
Weight (kg)	79 (51-130)	80.4 (52.1-130.3)	p = 0.850	Independent

BMI = body mass index.

**Figure 1:**
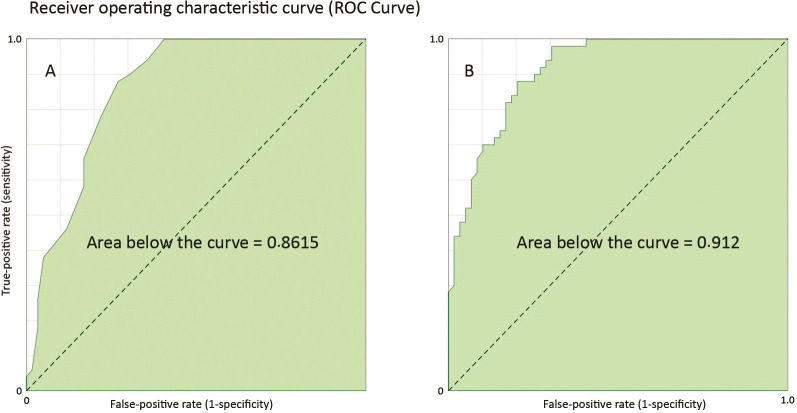
A) Receiver operating characteristic curve (ROC Curve) for analysis of total questionnaire score. B) ROC Curve for predictive model using individual question scores.

The preliminary analysis found that the majority of questions were significant, although the items “During or soon after pregnancy/breastfeeding, did you put on weight or notice changes to your thighs/legs, hips, buttocks, and/or arms?” (z score, p = 0.148) and “During the menopause, did you put on weight or notice changes to your thighs/legs, hips, buttocks, and/or arms?” (with no positive responses) did not attain the minimum number necessary for statistical analysis. They were therefore removed before creation of the predictive model using individual questions. Without these items, the receiver operating characteristic curve (ROC Curve) has an area of 0.912, which enabled construction of the mathematical predictive model (Figure 1B).

The total number of points summed (total score) was positively correlated with lipedema diagnosis (Pearson’s correlation p < 0.001 and chi-square p < 0.001), with odds ratio (OR) of 1.434 and standard error of 0.094 (constant of 0.046 and standard error of 0.028). The area under the ROC curve for total score is 0.8615 (Figure 1A). The mean time taken to complete the questionnaire was 3 minutes and 38 seconds, with a 100% rate of fully completed questionnaires.

### Prediction model

The formula for calculating the probability of lipedema using total score uses a total score coefficient of 0.361 and a constant of -3.075, as follows:

$${\left(e^{-(coef\;total\;scor\;+cons\tan t)}+1\right)}^{-1}$$

**Figure 2:**
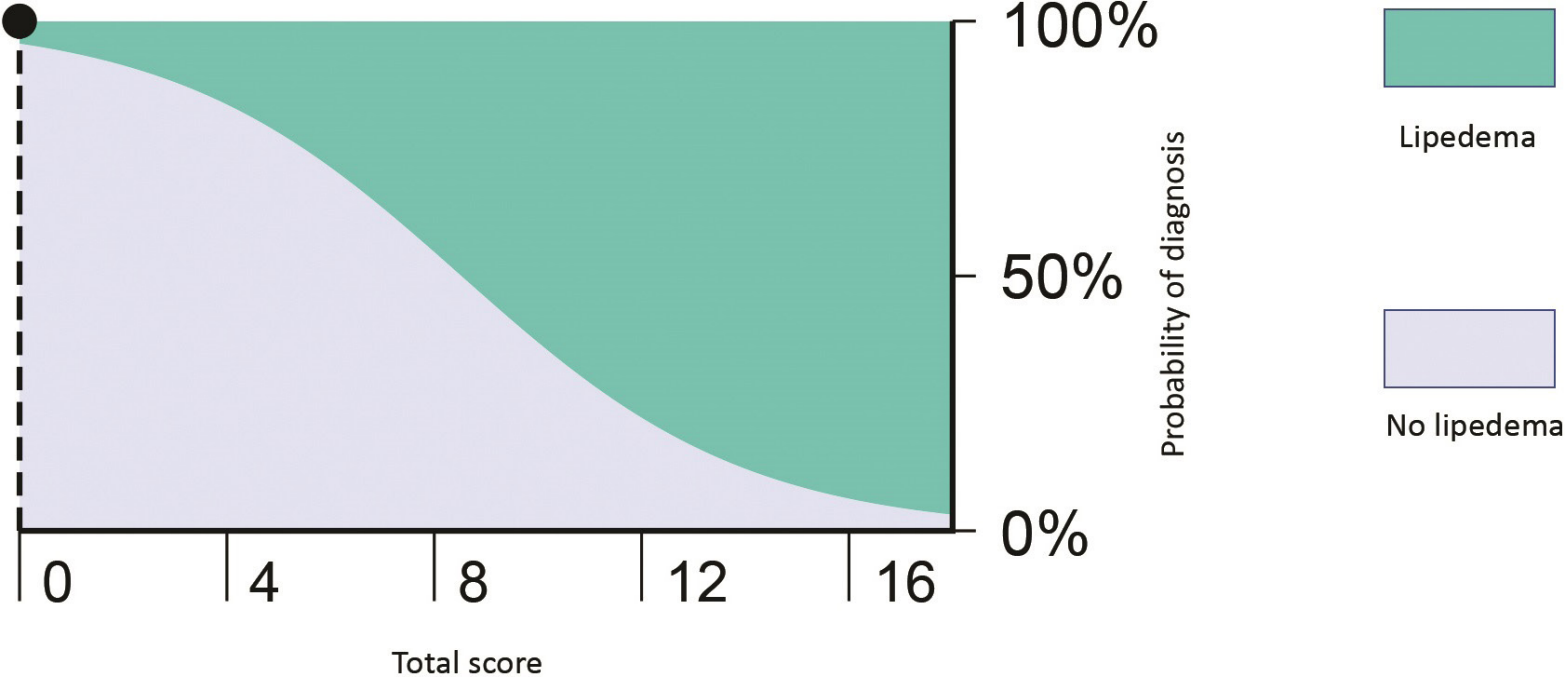
Model for prediction of lipedema diagnosis based on the total score.

This formula can be used to plot the model for predicting lipedema diagnosis (Figure 2).

The formula for calculating the probability of lipedema using the scores for each individual question employs a different coefficient for each question included (Table 3) as follows:

**Table 3:** Multivariate analysis of the influence of individual questions on lipedema diagnosis.

**Question**	**Coefficient**	**Odds ratio**	**Standard error**	**Rank by significance**
Do you feel that there is something wrong with your legs, but don’t know what it is? (p1)	1.465	4.328	1.699	1
Is the lower part of your body larger and disproportional compared to your trunk/upper body? (p2)	-0.092	0.912	0.494	5
Do you have problems losing weight, particularly from the lower part of your body? (p3)	0.437	1.548	0.702	4
During puberty, did you put on weight, primarily on the thighs/legs, hips, or arms? (p4)	-0.361	0.697	0.363	8
During or soon after pregnancy/breastfeeding, did you put on weight or notice changes to your thighs/legs, hips, buttocks, and/or arms? (p5)	-	excluded	excluded	
During the menopause, did you put on weight or notice changes to your thighs/legs, hips, buttocks, and/or arms? (p6)	-	excluded	excluded	
Do your legs hurt? (p7)	0.654	1.924	0.672	2
Do you have swollen legs? (p8)	0.458	1.58	0.744	3
Do your legs or arms bruise easily?? (p9)	-0.355	0.701	0.298	6
Constant	-3.555	0.029	0.022	

$${\left(e^{-(coef\;p1+coef\;p2+coef\;p3+coef\;p4+coef\;p7+coef\;p8+coef\;p9+cons\tan t)+1}\right)}^{-1}$$

This formula can be used to plot an individualized model for predicting lipedema.

## DISCUSSION

Lipedema is greatly underdiagnosed because there are no definitive and simple laboratory tests, imaging exams, or genetic tests^13^ and because it is easily confused with other diseases such as lymphedema, gynecoid obesity, and lipohypertrophy.^14^ Failure to correctly diagnose lipedema patients delays treatment of their disease, enabling it to progress.^13^ Lipedema symptoms and complaints may be considered subjective, particularly during the earlier phases, and are confused with other diseases that are more frequently seen in vascular clinics, such as chronic venous insufficiency, obesity, and lymphedema, revealing a need for standardization to increase the objectivity of diagnosis of this disease. It is therefore important to develop and validate instruments that can be used to assess the clinical impact of lipedema and facilitate definitive diagnosis.

Although the questions “During or soon after pregnancy/breastfeeding, did you put on weight or notice changes to your thighs/legs, hips, buttocks, and/or arms?” and “During the menopause, did you put on weight or notice changes to your thighs/legs, hips, buttocks, and/or arms?” did not attain statistical significance, we believe that the low number of individuals who had experienced these specific situations may have impacted on the scores for these questions, but not on the overall results of the questionnaire, which achieved good performance both for the individual model based on the scores for each question (area under the ROC curve = 0.912) and for the model based on total score (area under the ROC curve = 0.8615). These models can be interpreted as offering a high probability of correct diagnosis for distinguishing between patients with and without lipedema, at 91.2% and 86.15%, respectively.^15^ Notwithstanding, these questions are clinically relevant and may have a positive impact on the proposed tool, increasing its sensitivity and specificity. Therefore, we should continue to extend these analyses using larger samples. The statistical method used is useful for assessing sensitivity, but not necessarily specificity. In view of this, the questionnaire can be considered useful for preliminary assessment, but not for definitive diagnosis.

While the present study found evidence that the questionnaire has excellent predictive properties for lipedema diagnosis, correlating self-reported symptoms with lipedema diagnosis, we should remember the cyclical nature of lipedema symptoms^2^ which could discretely alter the results at different times, although the questions were designed to minimize this effect.

This tool was developed for screening for symptoms and to increase treating physicians’ diagnostic suspicion but should not be relied upon for definitive diagnosis. Proceeding with prospective assessments after this study should yield greater clinical applicability for the tool.

## CONCLUSIONS

The lipedema screening questionnaire is a practical instrument that is quick and easy to administer and can be used in our population for preliminary identification of patients who possibly have lipedema. This increases the level of suspicion when taking patient history and conducting a physical examination. Additional studies are still needed to assess the instrument as a support for definitive lipedema diagnosis and its correlations with other clinical features.
