# Supplementary material for: Development of a questionnaire and screening model for lipedema
Source: J Vasc Bras. 2020 Dec 11;19:e20200114. doi: 10.1590/1677-5449.200114 (PMC8218007; doi:10.1590/1677-5449.200114)
Supplement: Translation

# Translation: Criação de questionário e modelo de rastreamento de lipedema

**Authors:** Alexandre Campos Moraes Amato, Fernando Campos Moraes Amato, Daniel Augusto Benitti, Lorena Guimarães Lima Amato

**Affiliations:** 1 Universidade Santo Amaro – UNISA, São Paulo, SP, Brasil.; 2 Amato - Instituto de Medicina Avançada, São Paulo, SP, Brasil.; 3 Medical Valens Center, Campinas, SP, Brasil.; 4 Universidade Nove de Julho – UNINOVE, São Paulo, SP, Brasil.

**Keywords:** questionários, lipedema, obesidade, linfedema

## Abstract

**Contexto:**

O lipedema é muito subdiagnosticado e faltam ferramentas auxiliares diagnósticas de baixo custo. Baseado em um questionário de avaliação sintomática, criamos e validamos um questionário de rastreamento do lipedema.

**Objetivos:**

Os objetivos do trabalho foram a identificação de perguntas clínicas relevantes, a elaboração de questionário de rastreamento e a criação de modelo de predição do lipedema.

**Métodos:**

Um questionário simplificado foi criado e aplicado em um grupo de pacientes com e sem lipedema, sendo avaliada a probabilidade de acerto no diagnóstico.

**Resultados:**

Os 109 pacientes que responderam ao questionário eram do sexo feminino e as questões foram compreendidas. O modelo preditivo com perguntas individuais mostrou excelente probabilidade de acerto, de 91,2%, e o modelo preditivo com somatória de pontos também teve boa probabilidade de acerto, de 86,15%.

**Conclusões:**

O questionário de rastreamento do lipedema é um instrumento prático, de fácil e rápida aplicação, que pode ser utilizado em nossa população para a identificação de possíveis pacientes com lipedema, aumentando o nível de suspeição no momento da anamnese e exame físico.

## INTRODUÇÃO

O lipedema, descrito em nosso meio por Moraes IN como lipofilia membralis^1^, é caracterizado pela deposição de gordura anormal em glúteos, pernas e membros superiores bilateralmente, que pode ser acompanhada por edema ortostático em mulheres^2^ pós-puberdade. Casos anedóticos foram publicados em homens^3^. Embora a fisiopatologia do lipedema seja até hoje pouco compreendida, tem alta prevalência na população, explanada pelo seu aspecto genético e sua influência hormonal em sintomas inflamatórios cíclicos^2^. Foi descrito como processo inflamatório de baixo grau com acometimento do sistema linfático e gorduroso^2^, corroborado por recente trabalho de Wanshu Ma et al.^4^, sendo que essas características proporcionam sintomas típicos, muitas vezes mal interpretados, além da deposição de gordura. Sendo assim, apesar de condição distinta, é frequentemente confundido com doenças mais frequentemente diagnosticadas, como a obesidade e o linfedema^5^^,^^6^. O lipedema é uma doença de alta prevalência, porém de baixo reconhecimento e diagnóstico, sendo frequentemente subdiagnosticado ou confundido com obesidade ginecoide, linfedema ou mesmo insuficiência venosa^2^. Medidas conservadoras das estimativas da prevalência de lipedema na população em geral variam de 0,06 a 11%^5^^,^^7^.

O diagnóstico do lipedema é essencialmente clínico, definido pela desproporção simétrica do acúmulo de gordura nas extremidades inferiores acompanhada por queixas de edema ortostático^5^, frequentemente acompanhada por dor. Os pés são poupados desse aumento de tamanho exceto no estágio avançado de lipolinfedema, no qual o edema do pé ocorre secundariamente à insuficiência linfática inexistente em estágios anteriores^8^^,^^9^. O edema que poupa os pés é sinal importante para distinguir o lipedema da obesidade comum e linfedema. Como não há marcadores laboratoriais específicos para o lipedema, excetuando-se a recente promessa do fator 4 plaquetário^4^, o diagnóstico clínico requer alto nível de suspeição pelo médico assistente. Um questionário específico para o lipedema, elaborado na Alemanha^10^ e traduzido por Amato et al.^11^ baseou-se no questionário de qualidade de vida para pacientes com doenças linfáticas^11^^,^^12^ e foi adaptado para incluir 15 critérios autoavaliados por escala analógica de 0 a 10, sendo considerada uma escala sintomática do lipedema e não validada para uso como critério diagnóstico ou de rastreamento.

Tendo em vista a rara disponibilidade de instrumentos objetivos para o rastreamento do lipedema no Brasil e no mundo e que dados referentes à qualidade de vida são importantes para a seleção e a interpretação da evolução clínica, justifica-se este estudo tendo como objetivo identificar perguntas relevantes e criar um modelo matemático de predição, com o intuito de elevar o nível de suspeição pré-avaliação.

## MÉTODOS

O processo de identificação e simplificação de perguntas relevantes e passíveis de autoavaliação iniciou-se por meio da avaliação do questionário sintomático traduzido e da sua adaptação em forma (Tabela 1). O questionário foi convertido para versão digital *on-line* com *software* seguro e apropriado para desenvolvimento e análise de questionários (SurveyMonkey, Califórnia, EUA) e foi aplicado em 109 voluntárias, portadoras ou não de lipedema diagnosticado previamente em consulta médica, mas autorreportado no questionário. O estudo foi aprovado pelo Comitê de Ética (número 3.268.401).

**Tabela 1:** Versão final do questionário de rastreamento do lipedema.

Questionário de rastreamento do lipedema			
Pergunta	Resposta	Escore	Valor de p
Você sente que tem algo errado nas suas pernas, mas não sabe o que? (p1)	Sim, minhas pernas são grandes, parecem colunas/tronco de árvore e tenho gordura no tornozelo.	3	< 0,001
	Sim, minhas pernas são maiores (comparativamente) que o resto do meu corpo.	2	
	Sim, minhas pernas são grandes e proporcionais ao resto do meu corpo.	1	
	Não, minhas pernas estão bem.	0	
A parte de baixo do corpo é maior e desproporcional ao tronco/parte superior do corpo? (p2)	Sim, a parte de baixo do meu corpo é nitidamente desproporcional à parte de cima/tronco. O tamanho da minha calça é pelo menos 3 vezes maior que o tamanho de minha camisa.	2	< 0,001
	Sim, meu corpo inferior é discretamente desproporcional ao meu tronco/parte de cima. O tamanho da minha calça é de 1 a 2 vezes maior que o tamanho de minha camisa.	1	
	Não, a parte de cima do meu corpo/tronco é maior que a parte de baixo do meu corpo. O número do tamanho da minha camisa é maior que o número da minha calça.	0	
	Não, a parte de cima e de baixo do meu corpo são proporcionais. O número da minha camisa e calça são os mesmos.	0	
Você tem problema para perder peso, principalmente a parte de baixo do corpo? (p3)	Sim, não consigo perder peso por nada que eu faça, principalmente nas minhas coxas/pernas, quadril e/ou braços, que parecem sempre aumentar.	2	< 0,001
	Sim, tento muito e só pareço perder peso no tronco/parte de cima do corpo, frequentemente excluindo braços.	1	
	Não, com dieta e exercício consigo perder peso. O peso/gordura parece sair proporcionalmente do meu corpo inteiro	0	
	Não, não tenho problema de peso ou dificuldade para perder peso. Normalmente tenho um peso estável.	0	
Durante a puberdade, você ganhou peso principalmente nas coxas/pernas, quadris, nádegas ou braços? (p4)	Sim, ganhei muito peso durante a puberdade, principalmente nas minhas coxas e pernas, quadris, nádegas e/ou braços	2	< 0,001
	Sim, ganhei algum peso na minha puberdade. As coxas/pernas, quadris, nádegas e/ou braços pareciam ficar maior que outras partes do meu corpo.	1	
	Não, ganhei algum peso durante a puberdade que foi distribuído no corpo inteiro e não somente nas coxas/pernas, quadris, nádegas e braços.	0	
	Não, ganhei um pouco de peso, mas perdia facilmente com dieta e exercício.	0	
	Não, eu não ganhei peso. Meu peso foi bem estável durante a puberdade.	0	
Durante ou logo após a gravidez/amamentação, você ganhou peso ou teve mudança nas suas coxas/pernas, quadris, nádegas e/ou braços? (p5)	Sim, ganhei muito peso (> 23kg), principalmente nas coxas/pernas, quadris, nádegas e/ou braços durante ou logo após a gestação e/ou amamentação.	2	0,148
	Sim, ganhei entre 16-23kg, um pouco nas coxas/pernas, quadris, nádegas e/ou braços logo após a gestação e/ou amamentação.	1	
	Não, ganhei peso normalmente (11-15kg), nenhum peso extra foi localizado nas coxas/pernas, quadris, nádegas e/ou braços durante ou logo após a gestação e/ou amamentação.	0	
	Não, ganhei menos peso do que o esperado ou perdi peso durante ou logo após a gestação e/ou amamentação.	0	
	Não aplicável, não tive gestação.	0	
Durante a menopausa, você ganhou peso ou teve mudança nas suas coxas/pernas, quadris, nádegas e/ou braços? (p6)	Sim, ganhei bastante peso, e minhas coxas/pernas, quadris, nádegas e/ou braços cresceram.	2	-
	Sim, ganhei peso, e minhas coxas/pernas, quadris, nádegas e/ou braços cresceram um pouco.	1	
	Não muito, ganhei um pouco de peso, mas foi distribuído igualmente no meu corpo todo ou na minha barriga.	0	
	Não, não ganhei peso.	0	
	Não aplicável, ainda não passei pela menopausa.	0	
Suas pernas doem? (p7)	Sim, minhas pernas são muito sensíveis. São dolorosas ou sinto queimando mesmo quando não são tocadas.	3	< 0,001
	Sim, minhas pernas são dolorosas e incômodas com qualquer toque.	2	
	Às vezes, minhas pernas doem ao pressionar ou ficar muito tempo de pé.	1	
	Não, minhas pernas não doem.	0	
Você tem inchaço nas pernas? (p8)	Sim, minhas pernas parecem inchadas quase o tempo todo. Ficam piores no calor e quando úmido, e o inchaço não melhora ao elevar as pernas. O inchaço é igual dos dois lados.	2	0,001
	Sim, minhas pernas frequentemente parecem inchadas, mas às vezes melhoram com elevação. O inchaço ocorre dos dois lados.	1	
	Não, minhas pernas quase não incham, incham pouco no calor ou no tempo úmido ou somente após viagem de avião ou no período menstrual, mas rapidamente voltam ao tamanho normal, principalmente quando elevo as pernas. E/Ou somente uma das pernas incham.	0	
	Não, eu raramente sinto inchaço nas pernas.	0	
Suas pernas ou braços formam hematomas facilmente? (p9)	Sim, minhas pernas e braços formam hematomas muito facilmente, eu nem percebo onde consigo eles.	2	0,002
	Sim, minhas pernas e braços formam hematomas às vezes, com um contato mínimo.	1	
	Não formo hematomas facilmente.	0	

### Diagnóstico do lipedema

O diagnóstico do lipedema ainda é eminentemente clínico. Foram utilizados os seguintes critérios, por avaliador experiente, para a classificação do grupo portador da doença: história clínica sugestiva em mulheres após a puberdade; depósito simétrico bilateral de gordura abaixo do quadril, poupando os pés (sinal de Stemmer negativo); edema não depressível (sinal de Godet negativo), resistente à elevação dos membros; áreas afetadas dolorosas, sensíveis à palpação; e aumento de fragilidade capilar, com aparecimento espontâneo de equimoses^2^^,^^3^^,^^5^^,^^6^^,^^8^^,^^9^.

### Aplicação dos questionários

Na fase de validação *on-line* do questionário, as voluntárias de um grupo específico de portadoras e não portadoras de lipedema se propuseram a responder ao questionário de forma informatizada, sem auxílio externo e preenchendo todos os dados de forma livre. A técnica de amostragem foi não probabilística por conveniência, sendo que os sujeitos foram convidados a participar tardiamente após avaliação em ambulatório de cirurgia vascular. Foram incluídas mulheres, com idade maior que 18 anos, atendidas por qualquer queixa. Foram excluídos indivíduos do sexo masculino, que não assinaram termo de consentimento, que tinham condições arteriais ou venosas graves associadas e que não falassem ou compreendessem a língua portuguesa.

O estudo teve como objetivos primários a elaboração de um questionário simples, de rápido preenchimento, para rastreamento do lipedema. Secundariamente, foi criado modelo matemático de predição do lipedema baseado no questionário criado.

### Análise estatística

Após a verificação manual da consistência dos dados, foi feita a análise estatística. O método estatístico utilizado foi descritivo e de frequências. A correlação entre as variáveis do questionário foi avaliada por correlação de Spearman e teste de Shapiro-Wilk. O total de pontos foi avaliado pelo coeficiente de correlação de Pearson. A análise estatística foi realizada com auxílio do teste *t* de Student. Assumimos para as correlações um nível de significância estatística de p < 0,05. Os *softwares* utilizados para a análise dos dados foram o Excel (Microsoft, Novo México, EUA) e Wizard 1.9.40 (Evan Miller, Massachusetts, EUA).

O presente estudo segue as normas do Conselho Nacional de Saúde, referentes à resolução n˚ 196/96 sobre pesquisa envolvendo seres humanos. Também segue a declaração de Helsinki e foi aprovado pelo Comitê de Ética em Pesquisa da Plataforma Brasil, sob protocolo CAAE: 09590919.6.0000.0081.

## RESULTADOS

Cento e nove indivíduos responderam ao questionário com o conhecimento do diagnóstico prévio. As questões foram compreendidas por todos os entrevistados. Nenhuma questão foi considerada não aplicável. A proporção entre pacientes com e sem lipedema foi uniforme (z-score, p = 0,389) (Tabela 2).

**Tabela 2:** Caracterização da amostra.

	**Lipedema**	**Sem lipedema**	**Qui-quadrado**	**Resultado**
n	59	50	p = 0,389	Uniforme
Idade (anos)	42 (19-77)	45,7 (24-79)	p = 0,165	Independente
IMC (kg/cm^2^)	29,68 (20,4-46,2)	29,1 (20,4-45,6)	p = 0,312	Independente
Altura (cm)	163,7 (148-183)	165 (139-183)	p = 0,113	Independente
Peso (kg)	79 (51-130)	80,4 (52,1-130,3)	p = 0,850	Independente

IMC = índice de massa corporal.

A análise preliminar evidenciou significância na maioria das perguntas, sendo que as perguntas “Durante ou logo após a gravidez/amamentação, você ganhou peso ou teve mudança nas suas coxas/pernas, quadris, nádegas e/ou braços?” (z-score, p = 0,148) e “Durante a menopausa, você ganhou peso ou teve mudança nas suas coxas/pernas, quadris, nádegas e/ou braços?” (sem respostas positivas) não atingiram número mínimo necessário para o cálculo estatístico. Por isso, foram retiradas apenas da criação do modelo de predição por perguntas individuais. Sem elas, a curva característica de operação do receptor (*ROC Curve*) tem área de 0,912, o que permitiu a criação de modelo matemático preditivo (Figura 1B).

**Figura 1:** A) Curva característica de operação de receptor (*ROC Curve*) para análise da somatória dos pontos do questionário. B) *ROC Curve* para modelo de predição com perguntas individuais.

O total de pontos somados (escore somado) tem correlação positiva com o diagnóstico de lipedema (correlação de Pearson, p < 0,001 e qui-quadrado, p < 0,001), com *odds ratio* (OR) de 1,434 e erro padrão de 0,094 (constante de 0,046 e erro padrão de 0,028). A curva ROC para o total de pontos tem área de 0,8615 (Figura 1A). O tempo médio para resposta do questionário foi de 3 minutos e 38 segundos, com taxa de preenchimento completo de 100%.

### Modelo de predição

A fórmula para calcular a probabilidade do lipedema com os pontos totais requer o coeficiente dos pontos totais 0,361 e a constante de -3,075:

$${\left(e^{-(coef\;pontos\;totais\;+cons\tan te)}+1\right)}^{-1}$$

E permite criar modelo preditivo de diagnóstico do lipedema (Figura 2).

**Figura 2:** Modelo de predição do diagnóstico do lipedema baseado na somatória de pontos das respostas.

A fórmula para calcular a probabilidade do lipedema com perguntas individuais requer os coeficientes de cada pergunta utilizada (Tabela 3):

**Tabela 3:** Avaliação multivariável da influência das perguntas no diagnóstico do lipedema.

**Pergunta**	**Coeficiente**	***Odds ratio***	**Erro padrão**	**Hierarquia na significância**
Você sente que tem algo errado nas suas pernas, mas não sabe o que? (p1)	1,465	4,328	1,699	1
A parte de baixo do corpo é maior e desproporcional ao tronco/parte superior do corpo? (p2)	-0,092	0,912	0,494	5
Você tem problema para perder peso, principalmente a parte de baixo do corpo? (p3)	0,437	1,548	0,702	4
Durante a puberdade, você ganhou peso principalmente nas coxas/pernas, quadris, nádegas ou braços? (p4)	-0,361	0,697	0,363	8
Durante ou logo após a gravidez/amamentação, você ganhou peso ou teve mudança nas suas coxas/pernas, quadris, nádegas e/ou braços? (p5)	-	retirado	retirado	
Durante a menopausa você ganhou peso ou teve mudança nas suas coxas/pernas, quadris, nádegas e/ou braços? (p6)	-	retirado	retirado	
Suas pernas doem? (p7)	0,654	1,924	0,672	2
Você tem inchaço nas pernas? (p8)	0,458	1,58	0,744	3
Suas pernas ou braços formam hematomas facilmente? (p9)	-0,355	0,701	0,298	6
Constante	-3,555	0,029	0,022	

$${\left(e^{-(coef\;p1+coef\;p2+coef\;p3+coef\;p4+coef\;p7+coef\;p8+coef\;p9+cons\tan te)+1}\right)}^{-1}$$

E permitem o cálculo individualizado do modelo de predição.

## DISCUSSÃO

O lipedema é muito subdiagnosticado devido à falta de teste de laboratório, exame de imagem ou teste genético definitivo e fácil^13^, sendo facilmente confundido com outras doenças como linfedema, obesidade ginecoide e lipohipertrofia^14^. A ausência do diagnóstico correto em pacientes com lipedema atrasa o tratamento da doença, permitindo a sua progressão^13^. Os sintomas e queixas do lipedema podem ser considerados subjetivos, principalmente nas fases mais precoces, e confundem-se com outras doenças muito frequentes no consultório vascular, como insuficiência venosa crônica, obesidade e linfedema, de modo que é necessária a padronização para aumentar a objetividade do diagnóstico dessa enfermidade. Por isso, é importante desenvolver e validar instrumentos que possam avaliar o impacto clínico do lipedema e auxiliar no diagnóstico definitivo.

Apesar de as perguntas “Durante ou logo após a gravidez/amamentação, você ganhou peso ou teve mudança nas suas coxas/pernas, quadris, nádegas e/ou braços?” e “Durante a menopausa, você ganhou peso ou teve mudança nas suas coxas/pernas, quadris, nádegas e/ou braços?” não demonstrarem significância estatística, acreditamos que o baixo número de indivíduos que passaram pelas situações específicas citadas nas perguntas pode ter interferido no resultado pontual dessas perguntas, mas não no resultado global do questionário, que apresentou boa performance tanto no modelo de avaliação individual de cada pergunta (área ROC = 0,912) como no modelo de avaliação de pontos totais (área ROC = 0,8615). Eles podem ser interpretados como tendo excelente probabilidade de acerto na distinção entre portadora ou não de lipedema, sendo 91,2% e 86,15%, respectivamente^15^. Entretanto, elas são perguntas clinicamente relevantes e podem ter impacto positivo na ferramenta proposta, aumentando a sensibilidade e a especificidade. Portanto, devemos continuar e aprofundar as análises com um público maior. O método estatístico utilizado é útil para avaliar a sensibilidade, mas não necessariamente a especificidade; por isso, o questionário proposto pode ser considerado útil para a avaliação preliminar e não para o diagnóstico definitivo.

Embora o presente trabalho tenha evidenciado excelente predição do questionário proposto para o diagnóstico de lipedema, correlacionando sintomas autoavaliados com o diagnóstico de lipedema, devemos considerar que a natureza cíclica dos sintomas do lipedema^2^ pode alterar discretamente os resultados em diferentes momentos, embora as perguntas tenham sido elaboradas para minimizar esse efeito.

A ferramenta foi desenvolvida para o rastreamento dos sintomas e o aumento da suspeição diagnóstica do médico assistente, não devendo ser utilizada para diagnóstico definitivo. Proceder a avaliação prospectiva a partir deste estudo levará a maior aplicabilidade clínica da ferramenta.

## CONCLUSÃO

O questionário de rastreamento do lipedema é um instrumento prático, de fácil e rápida aplicação, que pode ser utilizado na nossa população para a identificação preliminar de possíveis pacientes com lipedema. Isso aumenta o nível de suspeição no momento da anamnese e do exame físico. Ainda são necessários estudos adicionais para avaliar o instrumento para o auxílio definitivo no diagnóstico do lipedema e na correlação com outros aspectos clínicos.
